# The nexus of financial development, natural resource rents, technological innovation, foreign direct investment, energy consumption, human capital, and trade on environmental degradation in the new BRICS economies

**DOI:** 10.1007/s11356-022-20976-7

**Published:** 2022-05-31

**Authors:** Fortune Ganda

**Affiliations:** Department of Accounting, Faculty of Management Sciences, Walter Sisulu University, Butterworth Campus, Private Bag X3182, Butterworth, 4980 South Africa

**Keywords:** Financial development of financial institutions, Financial development of financial markets, Natural resource rent, Trade, Energy consumption, Human capital, Technological innovation, Foreign direct investment (FDI), Carbon emissions

## Abstract

Early periods of history have demonstrated that enhanced economic development is fostered in instances where natural resources are abundant, hence averting the resource curse. In this vein, accelerated economic advancement is driven by a rigorous and proficient financial sector that efficiently utilises and allocates the economy’s natural resources. A strong financial system that transforms resources into advantages rests on an advanced technological innovation base, superior human capital, distinct foreign direct investment, powerful trade, and sustainable energy consumption. While this paper investigates the nexus of these factors, the specific purpose of this research is to examine the interactive impact of financial development and natural resource rents on carbon emissions in the new BRICS economies for the duration of 1990 to 2019. The panel data generalised least squares (GLS) and the panel-corrected standard error (PCSE) techniques are adopted. The Dumitrescu and Hurlin technique is used to establish causality. The study found a U-shaped association between economic growth and emissions. The findings prove that the financial development of financial institutions and the financial development of financial markets’ relationships with emissions are significantly positive. Natural resource rents, energy consumption, and human capital create a significantly positive relationship with emissions (mostly just positive for technological innovation). Conversely, the connection involving trade and carbon emissions is significantly negative (but mostly just negative for FDI). The interaction (s) intervening financial development of financial institutions and financial development of financial markets with natural resource rent significantly lowers emissions, respectively. The interaction parameter (financial development of financial institutions, natural resource rent, and financial development of financial markets) mixed with trade significantly adds emissions (positively insignificant with energy consumption). Contrarily, this factor mixed with human capital and technological innovation, respectively, is significantly negative (just negative for FDI). The Dumitrescu–Hurlin panel Granger causality outcomes are also outlined.

**Supplementary Information:**

The online version contains supplementary material available at 10.1007/s11356-022-20976-7.

## Introduction

Climate change and global warming are disputably the principal threats to the sustainability of the world as anthropogenic activities persistently emit large amounts of carbon emissions (Abid et al. 2022). One of the debatable issues relates to which human practices produce high carbon emissions and hence, what financial development and natural resource rent attributes do they possess with one another?

Numerous studies on parameters that trigger carbon emissions have specialised in the impacts of diverse associations on environmental quality findings (Ganda 2022). For instance, there are discussions on whether country emissions are a result of FDI, human capital, trade, economic growth, and so forth (Shen et al. 2021; Sun et al. 2020; Dagar et al. 2021) or their rootedness in the global society, mirrored in its engagement in worldwide environmental interests’ institutions (Joshua and Bekun 2020; Yao et al. 2021). Some schools of thought have disputed these empirical study results for disregarding other parameters (Li et al. 2021; Huang et al. 2021). In that case, there are perceptions that carbon emissions are fundamentally the outcome of the interaction of various natural, financial, political, social, technological, and environmental systems that govern the balance of power involving different stakeholders.

As such, it is apparent that there is research yet to analyse how parameters such as financial development and natural resource rents combine by themselves or with other systems or variables to produce carbon emissions. In this vein, it can be spotlighted that increase in carbon emissions is an overly complicated challenge that cannot be examined using a single, independent framework. To tackle this challenge, it is imperative to have an improved comprehension of where and why pollution initiatives take place. As such, few studies have analysed the integrating and/or interaction perspectives regarding environmental quality. On that note, a better understanding of specific structural profiles and interactions of available economic attributes is critical to determining their possible environmental conduct.

All the BRICS economies rely on natural resource rents for economic advancement; hence, it is a vital part of the worldwide economic base. Natural resource rents are constituted of coal, forests, minerals, gas and oil. Howbeit, the link involving natural resource rents and carbon emissions has always produced inconclusive findings. For instance, Shen et al. (2021), Ibrahim and Ajide (2021), and Usman et al. (2022) demonstrate that the relationship is positive. Conversely, Shittu et al. (2021) and Zhang et al. (2021) illustrate that the association is negative. In cases where natural resource rents are improved to become environmentally compatible, the EKC is ultimately produced. In circumstances where they boost the economic growth of the economy, better standards of living in the country are realised. In this vein, it is critical to spotlight that the financial sector of the country adds to better natural environmental welfare. An inefficient and weak financial sector damages the natural environment as they normally allow non-green industries to acquire funds that are detrimental to the natural ecosystems. However, an efficient and strong financial sector provides environmental finance to industries that propel high energy efficiency, low pollution generation, secure green research and development contexts to realise better technological innovation, and also spur the integration of low-carbon technologies and skills for business growth. Thus, reduced financial expenses, increased investment scale, motivating spillover of knowledge, and/or technologies along with fostering total factor productivity are also a function of well organised financial development.

There are many underpinning motivations for why this paper used the new BRICS economies as the focus. Firstly, the issue of carbon emissions is a principal continuous concern for these governments along with environmental interest groups (Adedoyin et al. 2020). Thus, Wang and Zhang (2020) contribute that although in the previous 30 years, BRICS gross domestic product heightened from 416.4 billion in 1990 to 18 188.4 billion in 2018 (thereby adding 22% to worldwide gross domestic product), excessive emissions and natural environment deterioration have exacerbated. As such, research that has been done to promote carbon emissions reduction has generated contradictory results. In addition, numerous policies have been proposed, but these have been unable to give a consensus as environmental quality challenges have continued to worsen.

Moreover, the existing dearth of research results that takes into account the interactive influence of financial development and natural resource rents on carbon emissions in the BRICS is apparent. Interaction of diverse systems on a national economic base is a vital platform through which green interest groups and green parties can achieve goals earmarked to foster sustainable development. This study also fills this gap in the literature by ascertaining the causality of variables, along with interactive contexts of these parameters on environmental quality. Thus, this paper expands beyond current research by simulating the interactive influence of financial development and natural resource rents on carbon emissions while accounting for the effects of other important factors (economic growth, trade openness, human capital, FDI, energy consumption, and technological innovation).

Therefore, this study attempts to answer the following main research questions: What is the impact and/or causality of financial development measures on carbon emissions? How do natural resource rents influence and/or cause environmental quality? What is the interactive effect and/or causality of financial development measures and natural resource rents on environmental degradation? Does a threshold point exist beyond which the effect of the financial sector transforms its influence on the natural environment, that is, the financial environmental Kuznets curve (FEKC)? Is there a non-linear association involving economic growth and carbon emissions, that is, the environmental Kuznets curve (EKC)?

This research adds to empirical studies in the following manner. Firstly, this paper investigates the interactive effect of financial development and natural resource rents on environmental degradation in the new BRICS economies from 1990 to 2019 for the new BRICS economies. Therefore, the paper provides empirical studies on the natural environment by offering new evidence from the new BRICS economies — Brazil, Russian Federation, India, China, South Africa, Bangladesh, Uruguay, and the United Arab Emirates. Second, this research integrates the interaction parameter between natural resource rents and the two proxies of financial development in affecting environmental quality. In the same vein, the paper will use an advanced econometric causality technique — Dumitrescu and Hurlin to examine the causality of variables. Third, to the best knowledge of the author, no previous research has clearly and distinctly spotlighted the interactive roles of financial development and natural resource rents which are also imperative in describing the ongoing global debate involving growth and environmental quality.

Fourth, earlier research does not provide answers as to whether financial development and/or natural resource rents persistently improve the state of the natural environment. In that regard, the interactive role of such parameters is vital to offer decision-makers guides to incorporate policies, strategies, systems, and technologies in proportion to levels of financial development and natural resource rents. In this vein, the existence of such influences and their consequences is a pragmatic matter that merits cognisance. Fifth, this paper is the first-panel analysis of natural resource rents, financial development of financial institutions, financial development of financial markets, economic growth, trade openness, human capital, FDI, energy consumption, technological innovation, and carbon emissions in the new BRICS economies; and the outcomes offer major enlightenments for creating an efficacious green strategy and policy in the new BRICS countries. Finally, this paper utilises major complex econometric approaches for empirical scrutiny, using the panel data generalised least squares (GLS), panel-corrected standard error (PCSE) techniques and the Dumitrescu and Hurlin causality approach for empirical estimates.

The remainder of the research is arranged as follows. The “Literature review” section describes the recent explorations on the topic. The “Research data” section discusses the data and methodology of the paper. The “Results” section explains the study findings and discussion. The “Implications of the study” section reveals the implications of the paper. The “Conclusion” section concludes the research.

## Literature review

A growing number of studies have investigated the relationships on variables such as natural resource rents, financial development of financial institutions, financial development of financial markets, economic growth, trade openness, human capital, FDI, energy consumption, and technological innovation impacts on environmental quality across the globe. As such, Table 1 presents an analysis of more recent research on the subject.

**Table 1:** Empirical research and the findings

Author(s)	Country (s)	Period	Variables	Methodology	Result (s)
Shen et al. (2021)	China	1995–2017	Natural resources rent (NR); economic growth (GDP); green investment (GI); financial development (FD); energy use (EC); carbon emissions (CO_2_)	Cross-sectionally augmented autoregressive distributed lags (CS-ARDL) method	Energy use, financial development and natural resources rent are positively linked to emissions, whereas green investment is negatively associated
Wang et al. (2020)	G7 countries	1996–2017	Economic globalisation (EG); FD; Agriculture value-added (AD); NR; GDP; CO_2_	CS-ARDL	EG, FD, and NR contribute to more emissions. AD lessens carbon emissions
Huang et al. (2021)	USA	1995–2015	FD; NR; urbanisation (U); CO_2_	Quantile autoregressive distributed lagged model (QARDL) approach	In the long-run FD, NR and U add to high emissions
Li et al. (2021)	G7 countries	1980–2018	FD (Markets); FD (financial institutions); NR; trade (TR); gross capital formation (GCF); GDP	Panel pooled mean group/autoregressive distributed lag model (PMG/ARDL)	The resource curse is disapproved, and the resource blessing is proven for the long run in these countries. In the short run, NR has a weak influence on FD (markets)
Ibrahim and Ajide (2021)	BRICS	1996–2018	Non-renewable energy (NNE); FD; NR; TR; Regulatory quality (RQ); CO_2_	Augmented mean group (AMG) and common correlated effect mean group (CCEMG) approach	Coal and fuel production and use trigger emissions. Also, NR, FD, and RQ foster emissions, while TR motivated emission effects are not supported
Ling et al. (2021)	China	1980–2017	EG; FD; NR; GDP; CO_2_	The nonlinear autoregressive distributed lag (NARDL); cross-wavelet modelling framework	Positive shocks of EG and FD add to increased emissions. NR negative shocks have a positive influence on emissions
Usman et al. (2022)	8 Arctic economies	1990–2017	FD; EC; NR; EG; renewable energy (RE); NNE; GDP; CO_2_	Panel data simulations	NR, GDP, and NNE heighten emissions. FD and RE lower emissions
Sun et al. (2020)	7 emerging economies	1990–2017	FD; NR; Human capital (HC); TR; GDP	Westerlund Cointegration tests; AMG	FD negatively influences NR, thereby confirming the resource curse hypothesis. An increase in HC and TR stimulates FD. GDP positively connects with FD increase
Dagar et al. (2021)	OECD countries	1990–2018	RE; FD; NR; EG; GDP; ecological footprint (EF)	Dumitrescu and Hurlin panel Granger causality test; AMG and CCEMG	The interactive influence of EG and FD reduces environmental degradation. NR, NNE, and FD heighten pollution. The growth hypothesis is present between NR and environmental quality
Yao et al. (2021)	BRICS, next 11 economies	1995–2014	Energy efficiency (EE); FD; EF; FD; NR; corruption control (CC); TR; technological innovation (TI); industrialisation (I)	Generalised method of moments (GMM); data envelopment analysis (DEA)	FD spur EE by reducing CC. FD lowers EF. TI is a primary determinant of EE
Joshua and Bekun (2020)	South Africa	1970–2017	NR; GDP; coal consumption (CN)	Dynamic autoregressive distributed lag models (ARDL)	The test demonstrates long term equilibrium links involving EC, CN, NR, GDP and pollution
Shittu et al. (2021)	45 Asian countries	1990–2018	NR, GDP, EF, energy security	Instrumental variable 2-stage least square	NR is negatively related to EF. The EKC is not valid
Zhang et al. (2021)	Pakistan	1985–2018	NR, GDP, EF, HC, CO_2_	Dynamic autoregressive distribution lag (DARDL) approach	NR is negatively associated with EF and emissions. Human capital lowers emissions but triggers EF. The EKC is established
Jahanger et al. (2022)	73 developing countries	1990–2016	NR, GDP, TI, HC, FD, EF, CO_2_	Panel cointegration, ARDL	NR and GD heighten EF. TI lowers EF. EKC for EF is valid
Jiang et al. (2022)	China	2007–2015	Coal energy, gas energy, coke, diesel, other petroleum energy sources, CO_2_	Input–output, extended structural decomposition, energy utilisation models	The input system and energy system impacts possess restraining power on the extending of emissions from non-renewable energies
Usman and Balsalobre-Lorente (2022)	10 newly industrialised countries	1990–2019	NR, GDP, RE, FD, I, RE, CO_2_	AMG, CCEMG, PMG, Dumitrescu and Hurlin (D-H) non-causality test	RE and NR mitigate emissions. FD and I add more emissions. The conservation hypothesis is confirmed for NR and EF. The feedback hypothesis is confirmed for FD and EF
Balsalobre-Lorente et al. (2022)	Portugal, Ireland, Italy, Greece, and Spain	1990–2019	FDI, RE, U, economic complexity index (ECI), CO_2_	Dynamic ordinary least square (DOLS) estimator, Dumitrescu and Hurlin (D-H) non-causality test	The pollution haven hypothesis is valid. RE lowers emissions, but the U effect is the reverse

## Research methodology

This section of the research outlines the data and econometric instruments used in the study.

### Data

This paper investigates the nexus of financial development, natural resource rents, technological innovation, foreign direct investment, energy consumption, human capital and trade on environmental quality in eight BRICS countries from 1990 to 2019. These economies are, namely, Brazil, Russian Federation, India, China, South Africa, Bangladesh, Uruguay, and the United Arab Emirates. A complete analysis of the data used in the paper including the sources is presented in Table 2.

**Table 2:** Data attributes

Variable	Definition	Unit	Source
LogCO_2_	Carbon emissions	Metric tons per capita	Global Carbon Project
LogFIS	Financial development of financial institutions	A composite index of financial institutions in terms of depth, access and efficiency	IMF database
LogFM	Financial development of financial markets	A composite index of financial markets in terms of depth, access and efficiency	IMF database
LogGDP	Economic growth	GDP per capita (constant 2010 US$)	WDI database
LogNR	Natural resource rents	Total natural resource rents (% of GDP)	WDI database
LogEC	Energy consumption	kg of oil equivalent per capita	WDI database
LogTR	Trade openness	Percentage of GDP	WDI database
LogFDI	Foreign direct investment	Net inflows (percentage of GDP)	WDI database
LogPA	Technological innovation	Patent applications [non-residents + residents (PAR)]	WDI database
LogHC	Human capital	Human capital index per person	Penn World Tables 10.0

### Econometric approach

This section presents the econometric approaches adopted by the paper.

#### Paneldata generalised least squaresand panel-corrected standard error techniques

This research deploys the CIPS panel unit root procedure (Pesaran 2007) to assist cross-sectional dependence matters. The study also applies panel unit root tests and one form of cointegration test (Kao 1999). The survey also employs the panel data generalised least squares (GLS) and panel-corrected standard error (PCSE) econometric frameworks to ascertain association among the parameters. Various studies have used the GLS estimator (Le et al. 2020; Shah et al. 2022) and, hence, will be utilised to estimate the relationships included in the regression. As the variables are economic, it is highly probable that explanatory variables and the error may correlate. On that note, the ordinary least square is not an effective econometric process to adopt (since it is unreliable and biassed). Thus, the GLS approach is the most appropriate method in contexts of correlation involving the error term and independent parameters (Zhou et al. 2020). The presence of the economy-specific influence (of each nation) by itself affects the outcomes of the regression. As such, such impacts should be considered as ignoring them may potentially result in omitted variable bias challenges, thereby rendering the estimated parameters to generate inconsistent results. Therefore, this paper has to manage the challenges of autocorrelation and cross-sectional heteroskedasticity which justifies the integration of the GLS estimator. In addition, this paper uses the PCSE framework to verify and/or substantiate the findings produced by the GLS econometric technique (Danish 2019). This paper argues that while the findings of the GLS approach will be compared to the PCSE technique outcomes, these outputs should generally be consistent (supporting each other with minute variances).

Deploying the GLS and PCSE approaches, initially this paper examines the direct influence of financial development, natural resource rents, and other independent variables on environmental quality. Therefore, the initial framework of this paper is arranged in Eq. (1) below:

1 $${LogCO}_{2it}={ \lambda }_{i}{{+ \lambda }_{1}LogGDP}_{it}+{ \lambda }_{2}{LogGDP}_{it}^{2}+{{ \lambda }_{3}LogFIS}_{it}+{{ \lambda }_{4}LogFM}_{it}+{{ \lambda }_{5}LogNR}_{it}+{{ \lambda }_{6}LogEC}_{it}+{{ \lambda }_{7}LogHC}_{it}+{{ \lambda }_{8}LogTR}_{it}+{{ \lambda }_{9}LogFDI}_{it}+{{ \lambda }_{10}LogPA}_{it}+{\varepsilon }_{it....}$$

where *i* represents a country and *t* depicts the year. $\lambda$’s show the slopes of the independent parameters when:

- ${\lambda }_{1}$> 0 and ${\lambda }_{2}$ < 0, the EKC hypothesis is valid (inverted U-shape).
- ${\lambda }_{1}$< 0 and ${\lambda }_{2}$ > 0, the U-shaped form is valid.
- ${\lambda }_{1}$> 0 and ${\lambda }_{2}$ > 0, a monotonically increasing relationship is valid.
- ${\lambda }_{1}$< 0 and ${\lambda }_{2}$ < 0, a monotonically decreasing relationship is valid.

In Eqs. (2) and (3), this paper considers the interactive impacts of financial development and natural resource rents and includes other explanatory variables as regressors. This is shown below as

2 $$LogCO_{2it}=\lambda_i\;+\;\lambda_1LogGDP_{it}\;+\;\lambda_2LogGDP_{it}^2\;+\;\lambda_3LogFIS_{it}\;+\;\lambda_4LogFM_{it}\;+\;\lambda_5LogNR_{it}\;+\;\lambda_6LogEC_{it\;}+\;\lambda_7LogHC_{it}\;+\;\lambda_8LogTR_{it}\;+\;\lambda_9LogFDI_{it\;}+\;\lambda_{10}LogPA_{it}\;+\;\lambda_3LogFIS_{it\mathit\;\ast}\;\lambda_5LogNR_{it}\;+\;\lambda_4LogFM_{it\mathit\;\ast}\;\lambda_5LogNR_{it}\;+\;\varepsilon_{it}\;....$$

3 $${{{LogCO}_{2it}={ \lambda }_{i}+{{ \lambda }_{1}LogGDP}_{it}+{ \lambda }_{2}{LogGDP}_{it}^{2}+\lambda }_{3}LogFIS}_{it} +{{ \lambda }_{4}LogFM}_{it} +{{ \lambda }_{5}LogNR}_{it} +{{ \lambda }_{6}LogEC}_{it} +{{ \lambda }_{7}LogHC}_{it} +{{ \lambda }_{8}LogTR}_{it} +{{ \lambda }_{9}LogFDI}_{it} +{{ \lambda }_{10}LogPA}_{it}+ {{ \lambda }_{3}LogFIS}_{it}*{{ \lambda }_{5}LogNR}_{it} +{{ \lambda }_{4}LogFM}_{it}*{{ \lambda }_{5}LogNR}_{it} + {{ \lambda }_{3}LogFIS}_{it}*{{ \lambda }_{5}LogNR}_{it}*{{ \lambda }_{3}LogFIS}_{it}*{{ \lambda }_{6}LogEC}_{it}+ {{ \lambda }_{3}LogFIS}_{it}*{{ \lambda }_{5}LogNR}_{it}*{{ \lambda }_{3}LogFIS}_{it}*{{ \lambda }_{7}LogHC}_{it}+{{ \lambda }_{3}LogFIS}_{it}*{{ \lambda }_{5}LogNR}_{it}*{{ \lambda }_{3}LogFIS}_{it}*{{ \lambda }_{8}LogTR}_{it}+{{ \lambda }_{3}LogFIS}_{it}*{{ \lambda }_{5}LogNR}_{it}*{{ \lambda }_{3}LogFIS}_{it}*{{ \lambda }_{9}LogFDI}_{it}+{{ \lambda }_{3}LogFIS}_{it}*{{ \lambda }_{5}LogNR}_{it}*{{ \lambda }_{3}LogFIS}_{it}*{{ \lambda }_{10}LogPA}_{it}+ {\varepsilon }_{it.}$$

#### Dumitrescu and Hurlin causality approach

This paper also integrates the Granger non-causality test process (Dumitrescu and Hurlin 2012) to examine the robustness of the study’s findings. The Dumitrescu-Hurlin test is described by the following linear framework:

$${y}_{i,t}={\alpha }_{i}+{\sum\nolimits}_{k=1}^{K}{\gamma }_{i}^{(k)}{y}_{i,t-k}+{\sum\nolimits}_{k=1}^{K}{\beta }_{i}^{(k)}{y}_{i,t-k}+{\varepsilon }_{i,t}\cdots ,\cdots i=\mathrm{1,2},\cdots Nt=\mathrm{1,2},\cdots T$$

As such, *x* and *y* depict two stationary parameters earmarked for *N* individuals in *T* periods. ${\beta }_{i}$ = $\left({\beta }_{i}^{(1)},\dots ..,{\beta }_{i}^{k}\right)$^*t*^ as well as individual impacts ${\alpha }_{i}$ are comprehended to be fixed in the time dimension classification. As well, the lag orders of *K* are assumed to be the same for the whole cross-section of panel data. Moreover, autoregressive parameters ${\gamma }_{i}^{(k)}$, as well as ${\beta }_{i}^{(k)}$ that are the equation coefficients, are allowed to be varied across groups.

In this vein, this approach assumes that coefficients are varied across the cross-sections, but the conventional Granger test underpins that those coefficients are in fact homogenous for all individuals in the panelised data. The supported procedure adds cross-sectional plus time series perspectives, thereby empowering it to take issues of heterogeneity into consideration as a result of individual differences (environmental, economic, structural, political, social, institutional, and so forth). The Dumitrescu and Hurlin (2012) technique computes the individual Granger causality of a particular cross-section and estimates the mean individual test processes taking into account of a statistical $\overline{w }$ statistic, $\overline{z }$ standardized statistic, and the approximated standardized statistic $\tilde{z }$. As such, the null hypothesis *y* does not homogenously cause *x*, and the reverse is confirmed. Dumitrescu and Hurlin (2012) contributes that the $\overline{w }$ statistic is employed in instances where *T* is found be less than *N*, whereas the $\overline{z }$ statistic is deployed in situations where *T* is more than *N*. Therefore, in this research, *T* (29 years) is more than *N* (8 countries) so the $\overline{z }$ statistic is supported.

## Results

This section demonstrates the study findings of the econometric procedures explained in the “Research methodology” section.

Initially, Table 3 depicts the attributes of data employed in this paper. It is noted that technological innovation has the greatest maximum value, whereas foreign direct investment possesses the least minimum value among the considered parameters. Economic growth is characterised with the highest mean value, but financial development of financial markets is attributed with the lowest mean estimate. All parameters are negatively skewed implying that their respective distributions have a predominantly tail on the left side. The Kurtosis is greater than 3 for economic growth, financial development of financial markets, trade, foreign direct investment, and technological innovation. Hence, for these factors, their distributions are peaked, and they have thick tails.

**Table 3:** Data features

Variable	Min	Std. Dev	Max	Mean	Skewness	Kurtosis
LogCO_2_	− 0.8664	0.6004	1.5523	0.5023	− 0.2934	2.3527
LogGDP	0	0.5744	5.0080	3.9252	− 1.1207	10.9193
LogFIS	− 0.7767	0.1707	− 0.1309	− 0.4388	− 0.0269	2.0783
LogNR	− 0.7137	0.5198	1.5649	0.5146	− 0.0322	2.2952
LogFM	− 2.1689	0.4484	− 0.1370	− 0.6154	− 1.3159	4.0644
LogEC	0	1.3426	4.0854	2.5026	− 1.0473	2.6782
LogHC	0.1652	0.0904	0.5359	0.3628	− 0.1916	2.3008
LogTR	0	0.4161	2.2474	1.5468	− 2.3275	10.0617
LogFDI	− 2.6028	0.6032	1.0960	0.0386	− 1.6327	6.6918
LogPA	0	1.4420	6.1881	3.4841	-0.9872	3.7770

To implement the GLS, PCSE, and the Dumitrescu and Hurlin causality approach, it is vital for the process to ensure that they are I (1), that is, the series are stationary after the first difference. On that note, this paper considers three univariate unit root procedures. These are namely, the augmented Dickey–Fuller (ADF) suggested by Dickey and Fuller (1981), Lagrange multiplier test proposed by Hadri (2000), and the Im-Pesaran-Shin put forward by Im et al. (2003). As such, the findings in Table 4 show that the parameters are stationary in the first difference for at least two of these panel unit root tests for all these variables employed in this paper. Howbeit, the mixed outcomes reflected by the first-generation panel unit root tests possibly indicate existence of cross-sectional dependence (as unit root tests that do not permit for cross-sectional dependence encounter size misrepresentations).

**Table 4:** Panel unit test findings

	At level			At 1st difference		
Variable	Fisher ADF statistic	Hadri LM statistic	Im-Pesaran-Shin statistic	Fisher ADF statistic	Hadri LM statistic	Im-Pesaran-Shin statistic
LogCO_2_	2.5866***	47.4533***	0.4582	29.2741***	− 0.3378	− 7.3174***
LogGDP	− 1.3006	20.6903***	3.3498	4.9498***	2.8751***	− 3.1098***
LogFIS	0.4210	44.8053***	1.7745	41.0654***	0.4791	− 8.6709***
LogNR	0.8690	20.5761***	− 0.9230	33.8715***	− 1.1044	− 8.2146***
LogFM	1.0593	37.1762***	− 1.0338	22.0270***	1.0042	− 7.1593***
LogEC	− 2.4899	24.7953***	3.2666	31.2290***	0.2332	− 8.2728***
LogHC	18.4635***	50.0090***	2.8011	0.9791	31.6142***	− 1.4355*
LogTR	3.0210***	32.6876***	− 1.7046**	27.8065***	2.6182***	− 7.4344***
LogFDI	2.5827***	24.9231***	− 2.5365**	43.5268***	− 1.6419	− 8.7467***
LogPA	4.8806***	29.3605***	− 1.6763**	41.6910***	− 0.0504	− 8.6833***

***; **, and * indicate that the coefficients are significant at the 1%, 5%, and 10% level of significance, respectively

To examine the possible presence of cross-sectional dependence (CD) this paper considers the Pesaran (2004) CD test technique. In this context, Pesaran (2004) developed an ordinary test of error cross-sectional dependence founded on the mean pairwise association estimates of the ordinary least square residuals that were extracted from the standard ADF equation (Dickey and Fuller 1979) for each individual series. Hence, the Pesaran CD test null hypothesis is no presence of cross-sectional dependence, while the reverse is true for the alternative hypothesis. The findings of the test in Table 5 vigorously reject the null hypothesis which means that there is high dependence between parameters (the factors are cross-sectionally independent).

**Table 5:** Outlines of Pesaran CD test results and slope homogeneity finding

Variables	CD_Pesaran_ test (2004)	
	Statistic	*p* value
LogCO_2_	4.29	0.000
LogGDP	18.88	0.000
LogFIS	23.48	0.000
LogNR	15.24	0.000
LogFM	15.68	0.000
LogEC	28.77	0.000
LogHC	27.37	0.000
LogTR	15.96	0.000
LogFDI	13.39	0.000
LogPA	12.34	0.000
Slope-homogeneity — with LogCO_2_ as the dependent parameter		
Delta	9.830 ***	
Adj. Delta	12.367 ***	

[1] ***; **, and * indicate that the coefficients are significant at the 1%, 5%, and 10% level of significance, respectively

Another area of concern in panel data investigations is to determine if the slope coefficients are similar. Thus, this research uses the Pesaran and Yamagata (2008) approach an improved version than the original Swamy (1970) slope homogeneity test. The null hypothesis of slope homogeneity is investigated against the alternative hypothesis of heterogeneity. This paper’ outcomes reject slope homogeneity, thereby confirming that there is presence of heterogeneity of the slope coefficients for the sampled economies (permitting the deployment of econometric heterogenous panel methods).

In this section of the paper, the research investigates the stationary levels of parameters by employing the CIPS test (Pesaran, 2007) that takes into account cross-sectional dependence. Therefore, the null hypothesis conveying the unit root is analysed at odds with the alternative hypothesis which exhibits stationarity. Hence, if the test statistic is greater than the critical estimates, the null hypothesis is not accepted, and the reverse holds. Table 6 outlines the CIPS panel unit root outcomes. Thus, with the exception of economic growth, all variables show that they are stationary at their first difference for the constant and trend applications. In this vein, the series is to a greatest extent integrated of first order I(1) so the study can deploy co-integration to understand whether the parameters have a long-run association.

**Table 6:** CIPS test findings of unit roots

Variables	Level [constant and trend]	First difference [constant and trend]
LogCO_2_	− 2.461	− 4.715
LogGDP	− 1.003	− 2.294
LogFIS	− 2.716	− 5.513
LogNR	− 2.971	− 5.572
LogFM	− 2.379	− 4.677
LogEC	− 2.396	− 4.947
LogHC	− 2.119	− 2.946
LogTR	− 2.914	− 5.153
LogFDI	− 3.941	− 5.787
LogPA	− 3.086	− 5.474

[1] The critical values at 1%, 5%, and 10% are − 2.73, − 2.86, and − 3.1 respectively.

It is critical to examine the existence of cointegration among the parameters of this study. This paper utilised the panel cointegration approach proposed by Kao (1999) to scrutinise the existence of a long-term connection between carbon emissions, natural resource rents, financial development of financial institutions, financial development of financial markets, economic growth, trade openness, human capital, FDI, energy consumption, and technological innovation for the dataset within the 7 BRICS economies. Kao (1999) ascertains the homogenous co-integration link by employing the Dickey-Fuller test and the augmented Dickey-Fuller test using the null hypothesis of no co-integration among the variables. The finding of outcomes in Table 7 regarding co-integration highlights that there is no co-integrating association using the Kao co-integration tests as the augmented Dickey-Fuller statistic is not significant at the 1%, 5%, and 10% level of significance.

**Table 7:** Kao cointegration test findings

Regression: Dep. Var	Test statistic	*t*-statistics	*p* value
LogCO_2_	Augmented Dickey-Fuller (*ADF*)	− 0.800116	0.2118

Table 8 illustrates the findings of the GLS econometric approach on a short-run basis since the co-integration tests did not confirm a long-run relationship. The findings of the PCSE regressions are only used for robustness purposes. On that note, it is apparent that there is a U-shaped relationship between economic growth and carbon emissions as shown in model 1 and model 3. Dkhili (2022) confirm the presence of the inverted U-shaped hypothesis (EKC) for the MENA countries over the period 1990 to 2018. Financial development of financial institution relationship with environmental quality is not significant in both model 1 (negative) and model 3 (positive). However, financial development of financial market link with carbon emissions is significantly positive in GLS framework 1 and 3. Anwar et al. (2021) research on 15 Asian countries spanning 1990 to 2014 also spotlights that financial development increase emissions.

**Table 8:** GLS and PCSE findings for models 1–4

Variable	GLS model 1	PCSE model 2	GLS model 3	PCSE model 4
LogGDP	− 0.7020*** (0.0900)	− 0.7128*** (0.0752)	− 0.5973*** (0.0804)	− 0.6238*** (0.0711)
LogGDP^2^	0.1506*** (0.0174)	0.1532*** (0.01458)	0.1287*** (0.0153)	0.1342*** (0.0135)
LogFIS	− 0.0937 (0.0943)	− 0.036 (0.0883)	0.0724 (0.1189)	0.1357 (0.1140)
LogNR	0.1027*** (0.0242)	0.1568*** (0.0301)	0.4635*** (0.0689)	0.4602*** (0.0718)
LogFM	0.0824** (0.0398)	0.1185*** (0.0428)	0.1866*** (0.0456)	0.1793*** (0.0484)
LogEC	0.0066 (0.0052)	0.0119* (0.007)	0.0107* (0.0057)	0.0175** (0.0082)
LogHC	2.0747*** (0.2798)	1.8852*** (0.2601)	2.6234*** (0.2567)	2.2308*** (0.2385)
LogTR	− 0.0562** (0.0253)	− 0.0542** (0.0232)	− 0.0503* (0.026)	− 0.0450** (0.0252)
LogFDI	− 0.0050 (0.0103)	− 0.0073 (0.0097)	− 0.0101 (0.0115)	− 0.0136 (0.0117)
LogPA	− 0.0021 (0.0074)	0.0009 (0.007)	− 0.0028 (0.0078)	0.0021 (0.0079)
LogFIS × LogNR	-	-	− 0.5334*** (0.1695)	− 0.446*** (0.1474)
LogFM × LogNR	-	-	− 0.3042*** (0.0804)	− 0.2564*** (0.0843)
Constant	0.1825 (0.1684)	0.2386 (0.1467)	0.0355 (0.1567)	0.1438 (0.1360)
Wald chi2(10)	585.60***		897.34***	
*R*.^2^		0.6598		0.7321
Observations	239	239	239	239

***, **, and * mean significant at 1%, 5%, and 10% significance level, respectively. Numbers in brackets are standard errors.

Moreover, the results of this study show that natural resource rents develop a positive and significant relationship with environmental degradation as shown in models 1 and 3. However, Shittu et al. (2021) study of 45 Asian resource-rich economies using an instrumental variable 2-stage least square approach posits that natural resource rent is negatively connected to environmental quality. Energy use is positively associated with carbon emissions in models 1 (not significant) and 3 (significant). Cheikh et al. (2021) study on the 12 MENA economies from 1980 to 2015 proves that emissions behave nonlinearly to energy use and income such that an inverted U-shaped trend for energy consumption effect on environmental quality is found. This paper also contributes that human capital show a positive and significant association with carbon emissions in the new BRICS setting. As such, by employing a fixed effect panel threshold model, Ganda (2021a) research on the old BRICS block spanning 2000 to 2018 adds that human capital lessens emissions when the threshold level is above 1.6375.

This research also expresses that trade is negative and significantly associated with carbon emissions in both models 1 and 3 at the 5% and 10% significance levels, respectively. In this vein, Leitão (2021) study on Portugal from 1970 to 2016 explains that trade adds to environmental improvements. This research also confirms that foreign direct investment and technological innovation connections with carbon emissions is just negative (model 1 and 3). Abid et al. (2022) exploration of the G8 economies between 1990 and 2019 expresses that FDI and technological innovation relationships to carbon emissions are significantly negative associated.

Other interesting findings have also been discovered in this research. Firstly, the interaction between financial development of financial institutions and natural resource rent significantly lowers environmental degradation. Secondly, the interaction involving financial development of financial markets and natural resource rent also significantly lessens carbon emissions. To confirm robustness of the results model 2 and model 4 depicts findings of the PCSE equations. It is evidenced that the PCSE results are in line with GLS results in an overwhelming manner except for only the magnitude (which is not also varied considerably) of the coefficients.

This paper further includes more interaction variables in the regression equations to further investigate relationships in Table 9. Thus, engaging findings are generated. Firstly, it is evident that the interaction term (financial development of financial institutions, natural resource rent, and financial development of financial markets) mix with trade significantly increases carbon emissions as shown in model 5. In case of energy consumption, this relationship is positively insignificant. Secondly, the interaction parameter (financial development of financial institutions, natural resource rent, and financial development of financial markets) mix with human capital and technological innovation, respectively, generates a negative and significant association with environmental quality. In the context of foreign direct investment, this link is just negative and insignificant. Third, key findings in model 7 demonstrate that financial development of financial markets and its squared estimate develop a significantly positive relationship with emissions. On that note, we find evidence of no financial environmental Kuznets curve (FEKC) hypothesis but a monotonic increasing connection involving financial development of financial markets and environmental degradation Table 10.

**Table 9:** GLS and PCSE results for models 5–8

Variable	GLS model 5	PCSE model 6	GLS model 7	PCSE model 8
LogGDP	0.0634** (0.0252)	0.0572*** (0.0266)	− 0.4687*** (0.0861)	− 0.5491*** (0.0843)
LogGDP^2^	-	-	0.1009*** (0.0156)	0.1116*** (0.0149)
LogFIS	0.1805 (0 0.1155)	0.3052*** (0.1093)	− 0.1611 (0.4608)	0.3999 (0.4983)
LogFIS^2^	-	-	− 0.3610 (0.4116)	− 0.0516 (0.4471)
LogNR	0.9925*** (0.1518)	1.1903*** (0.1685)	1.3214*** (0.1599)	1.5165*** (0.1838)
LogFM	0.1715*** (0.0456)	0.1555*** (0.0469)	0.6861*** (0.1223)	0.5295*** (0.1256)
LogFM^2^	-	-	0.2098*** (0.0550)	0.1414** (0.0555)
LogEC	0.0094 (0.0068)	0.0104 (0.0086)	0.0186** (0.0082)	0.0233** (0.0116)
LogHC	3.5350*** (0.2465)	3.1868** (0.2497)	2.7108** (0.2580)	2.2021*** (0.2418)
LogTR	− 0.1547*** (0.0575)	− 0.1379** (0.0621)	− 0.0516 (0.0612)	0.0552 (0.0662)
LogFDI	− 0.1548 (0.0575)	− 0.0062 (0.0136)	− 0.0159 (0.0156)	− 0.0281 (0.0173)
LogPA	0.0176 (0.0111)	0.0337*** (0.0106)	0.0267** (0.0114)	0.0119 (0.011)
LogFIS × LogNR	0.0386*** (0.0106)	− 1.0072*** (0.1976)	− 1.1058*** (0.254)	− 1.2636*** (0.2761)
LogFM × LogNR	− 0.7073*** (0.1446)	− 0.7435*** (0.1619)	− 1.0637*** (0.1521)	− 1.1824*** (0.1707)
LogFIS × LogFM × LogNR × LogFDI	− 0.0524 (0.0487)	− 0.0557 (0.0519)	− 0.0509 (0.0479)	− 0.051 (0.0476)
LogFIS × LogFM × LogNR × LogTR	0.2496* (0.1457)	0.2215 (0.158)	0.051 (0.1493)	− 0.1557 (0.156)
LogFIS × LogFM × LogNR × LogEC	0.0943 (0.0667)	0.0708 (0.0693)	0.0893 (0.0678)	0.0661 (0.0687)
LogFIS × LogFM × LogNR × LogHC	− 1.6017* (0.8165)	− 1.8223** (0.8749)	− 2.5869*** (0.8293)	− 2.6256*** (0.8143)
LogFIS × LogFM × LogNR × LogPA	− 0.3321*** (0.0633)	-0.3256*** (0.063)	− 0.1811** (0.0704)	− 0.1174* (0.0691)
Constant	-0.8922*** (0.1241)	-0.7212*** (0.1205)	-0.1049 (0.1994)	0.2109 (0.1831)
Wald chi2(10)	706.89***	-	1687.91***	-
R^2^		0.7195		0.8398
Observations	239	239	239	239

***, **, and * mean significant at 1%, 5%, and 10% significance level, respectively. Numbers in brackets are standard errors.

**Table 10:** Findings of pair-wise Granger causality tests between variables and emissions

Null hypothesis	$\overline{z }$ statistic	Lag order	Causality flow
LogCO_2_ does not homogenously cause LogGDP	6.6901***	1	LogCO_2_ → LogGDP
LogGDP does not homogenously cause LogCO_2_	5.9830	1	
LogCO_2_ does not homogenously cause LogFIS	4.9822*	2	LogCO_2_ $\leftrightarrow$ LogFIS
LogFIS does not homogenously cause LogCO_2_	4.9666*	2	
LogCO_2_ does not homogenously cause LogNR	1.2413	1	LogNR → LogCO_2_
LogNR does not homogenously cause LogCO_2_	4.4785***	1	
LogCO_2_ does not homogenously cause LogFM	0.7610	1	$\ne$
LogFM does not homogenously cause LogCO_2_	0.3706	1	
LogCO_2_ does not homogenously cause LogEC	3.0322	1	LogEC → LogCO_2_
LogEC does not homogenously cause LogCO_2_	3.9874**	1	
LogCO_2_ does not homogenously cause LogHC	5.1997**	1	LogCO_2_ → LogHC
LogHC does not homogenously cause LogCO_2_	5.0851	1	
LogCO_2_ does not homogenously cause LogTR	4.8534*	1	LogCO_2_ $\leftrightarrow$ LogTR
LogTR does not homogenously cause LogCO_2_	9.5902***	1	
LogCO_2_ does not homogenously cause LogFDI	5.0376**	2	LogCO_2_ $\leftrightarrow$ LogFDI
LogFDI does not homogenously cause LogCO_2_	3.0833*	2	
LogCO_2_ does not homogenously cause LogPA	2.1436	2	LogPA → LogCO_2_
LogPA does not homogenously cause LogCO_2_	3.2452*	2	
LogCO_2_ does not homogenously cause LogFIS × LogNR	0.8918	1	LogFIS × LogNR → LogCO_2_
LogFIS × LogNR does not homogenously cause LogCO_2_	4.7891**	1	
LogCO_2_ does not homogenously cause LogFM × LogNR	-0.6414	1	$\ne$
LogFM × LogNR does not homogenously cause LogCO_2_	2.1810	1	
LogCO_2_ does not homogenously cause LogFIS × LogFM × LogNR × LogFDI	5.3613**	2	LogCO_2_ → LogFIS × LogFM × LogNR × LogFDI
LogFIS × LogFM × LogNR × LogFDI does not homogenously cause LogCO_2_	0.2305	2	
LogCO_2_ does not homogenously cause LogFIS × LogFM × LogNR × LogTR	1.0456	2	LogFIS × LogFM × LogNR × LogTR → LogCO_2_
LogFIS × LogFM × LogNR × LogTR does not homogenously cause LogCO_2_	5.1680***	2	
LogCO_2_ does not homogenously cause LogFIS × LogFM × LogNR × LogEC	0.1813	1	LogFIS × LogFM × LogNR × LogEC → LogCO_2_
LogFIS × LogFM × LogNR × LogEC does not homogenously cause LogCO_2_	3.0019*	1	
LogCO_2_ does not homogenously cause LogFIS × LogFM × LogNR × LogHC	-0.4261	1	$\ne$
LogFIS × LogFM × LogNR × LogHC does not homogenously cause LogCO_2_	2.1954	1	
LogCO_2_ does not homogenously cause LogFIS × LogFM × LogNR × LogPA	0.3416	1	LogFIS × LogFM × LogNR × LogPA → LogCO_2_
LogFIS × LogFM × LogNR × LogPA does not homogenously cause LogCO_2_	2.5785*	1	

*** indicates estimates significant at 1%; ** outline numerical values significant at 5%; and * denotes estimates significant at 10%.

The GLS findings endorsed by the PCSE model produced study results about directional relationship involving the parameters although causality is not established. Therefore, this paper engages the Dumitrescu and Hurlin causality approach to determine the pair-wise Granger causality tests outcomes involving the factors and environmental degradation. This study finds a unidirectional causality relationship between carbon emissions and/or economic growth, and human capital. Ganda (2021b) describes that environmental quality causes human capital although the case of economic growth produces a one-way direction to emissions. In addition, this paper finds a one-way causality from natural resource rents, energy consumption, and technological innovation to carbon emissions. Ahmad et al. (2020) research of 22 emerging economies from 1984 to 2016 illustrates that technological innovation and natural resources considerably alter environmental degradation and vice versa.

This research also establishes a bidirectional causality association between carbon emissions and financial development of financial institutions, trade, and foreign direct investments. Zubair et al. (2020) study in Nigeria found a two-way effect of FDI on environmental quality between 1980 and 2018 Ponce and Alvarado (2019) analysis of 100 economies spanning 1980 to 2017 indicates a bidirectional connection between trade and environmental quality. As well, Sheraz et al. (2022) confirms that financial development and emissions depicts bidirectional causality links.

Moreover, the paper establishes more valuable findings from the interaction variables considered in the models. Firstly, the interaction factor consisting of financial development of financial institutions and natural resource rents develop a unidirectional causality relationship from this parameter to carbon emissions. Moreover, a one-way causality link is discovered running from the interaction term (financial development of financial institutions, financial development of financial markets, natural resource rents) with either FDI, trade, energy consumption, or technological innovation to environmental quality. A unidirectional causality association running from carbon emissions to the interaction parameter (financial development of financial institutions, financial development of financial markets, natural resource rents, and FDI) is ascertained.

## Implications of the study

There are various suggestions which can be made based on the study findings of the paper. Firstly, the study results do not show evidence of the EKC hypothesis. Instead, the paper show that there is a U-shaped connection involving economic growth and carbon emissions. In this case, heightening economic growth results in decreasing levels of carbon emissions and attains a specific threshold, beyond that heightening levels of income increase carbon emissions. Such contexts indicate that beyond a particular level of economic development, an additional increase of income can be realised at the cost of environmental damage. In situations where the industrial base of the country expands, more carbon emissions are produced. Hence, heightened production along with consumption increase degradation of the environment which shows that economic advancement generates detrimental effects on the natural environment. As such there should be green and environmental policies in the BRICS economic practices, especially industries so that they can reduce the level of carbon emissions by integrating environmentally compatible technologies and procedures. In this regard, change to green equipments and processes earmarked towards mitigating carbon emissions should be enhanced. Such initiatives promote sustainable economic advancement as they serve to ensure that the economy remains green and future generations can benefit from the protected environment. This is vital since the paper’s findings confirm that carbon emissions are actually causing economic growth, that is, economic development that is unsustainable.

Though there are mixed outcomes on the effect of financial development on environmental quality, the predominant outcomes confirm that a positive relationship is apparent. Thus, most study models show that both financial development of financial institutions and financial development of financial market increase and also spur carbon emissions in the sampled BRICS economies. Therefore, it is imperative to boost the functioning of both institutions and markets in the financial sector to support sustainable green growth. For instance, incorporation of green banks and green capital markets is a critical policy which will support generation of low-carbon economies. In addition, financial sectors in the BRICS should apportion financial capital to companies and projects that adopt environmentally friendly technologies and operations. At the moment, this study shows that existing technological innovation is causing degradation of the natural environment. In addition, decision-makers should allocate budgets to green and environmentally sustainable projects and investments. As well, policy strategists integrate green public reporting instruments which demands compulsory disclosure of company socially responsible practices to harness the possible green advantages of the financial sector. Thus, it is inevitable that once the financial sector is green, the governments can adopt these institutions and markets as regulatory platforms which foster green economy growth.

The findings of this study also demonstrate that natural resource rents increase in the BRICS economies and are also fostering heightened environmental damage. Moreover, this paper also depicts that current natural resource rents in the countries have also been discovered to cause environmental degradation. This scenario is possibly contributed owing to overutilisation of natural resources, thereby having detrimental influence on biological capacities of existing ecosystems. It is also apparent that human demand for earth’s ecosystems has exacerbated such that ecological deficits have been produced. While BRICS economies are rapidly expanding industrially, it is unavoidable that rise in natural resource extraction takes place coupled with unsustainable exploitation of non-renewable energy which increases both emissions and climate change. Therefore, it is critical for these economies to strengthen green policies that preserve the natural environment. For instance, natural resource regulatory systems and taxes enhance generation of environmentally friendly natural ecosystems, along with green compatible industrial and company processes. In this vein, revenues generated by utilisation of resources in production along with processing do not degrade the natural environmental quality. Besides encouraging people to give attention to natural environment policymakers should also create suitable legislations that heighten high adoption of green energy technologies. Renewable energy integration is vital since evidence of the study outcomes highlights that energy use is causing carbon emissions.

Furthermore, the findings of the paper demonstrate that the interaction between both financial development proxies (financial development of financial institutions and financial development of financial markets) with natural resource rent considerably reduces carbon emissions. Thus, while these variables independently increase emissions, their combined impacts improve quality of the natural environment in the BRICS. On that note, policymakers should integrate strategies that harness financial development and natural resource rents so that emissions continue to mitigate. For example, natural resource rents (forest, mineral, oil, coal, and natural gas) financial gains should be employed into the financial sector. In that case, they can be sustainably deployed to create an efficient green financial sector that supports productive and consumption activities that are using environmentally friendly initiatives, thereby ultimately doing away with even unsustainable natural resource rents (for example, oil).

Moreover, the interaction variable (financial development of financial institutions, natural resource rent and financial development of financial markets) combination with either human capital or technological innovation lessens emissions significantly. With regard to foreign direct investment, this relationship is just insignificantly negative. As such, it can be seen that human capital and technological innovation independent perspective significantly adds to more emissions in the sampled countries, but the mix with the interaction term improves the natural environment. This shows that there is need to create green human capital and green technological innovation in these economies so that emissions can further be minimised. The fact that human capital and technological innovation are currently not environmentally compatible is a cause for concern. In such instances, green education and green skill growth is recommended for long-run positive influence on the natural environment. Decision-makers should also promote green innovations along with doing away with different forms of red tapes which make it difficult to apply for green patents. In addition, encouraging green research and development is imperative in promoting energy efficiency and the environment. Energy use in this study appears to increase emissions; therefore, there should be increasing adoption of renewable energy technologies. Foreign companies, by FDI, required to be examined and screened based on whether they will effectively lower emissions in host countries.

This paper also establishes bidirectional causality between carbon emissions and financial development of financial institutions, plus FDI. Thus, this illustrates that this variable adds in promoting emissions through enhancing access to credit for institutions that do not support green economy generation objectives. As such, the financial sector should support green interest discounts, energy saving technologies in investment procedures. In addition, there should be introduction of green strategies and green policies which encourage low-carbon financing options. Causality test findings also demonstrate that the interaction between financial development of financial institutions and natural resource rents to carbon emissions exists. As well, a one-way causality link is shown running from the interaction variable (financial development of financial institutions, financial development of financial markets, natural resource rents) with either FDI, trade, energy consumption, or technological innovation to carbon emissions. These results show that these variable combinations in the economy are not harnessed by existing environmental and/or green policies adopted by policymakers at macroeconomic level. Thus, while some initiatives individually lower emissions, their integration with other components of the economy raises emissions. On that note, there is need to investigate networks and synergies within the whole economic system and develop green policies that enhance low-carbon environments on specific parts of the economic system. Furthermore, environmentally unfriendly units of the economy should be removed or improved in line with sustainability regulations.

## Conclusion

It is apparent that initiatives developed in the United Nations Framework Convention on Climate Change in recent years and the engagement of almost two hundred countries to the Paris summit induce matters such as global warming and carbon emissions at the centre of global topical debates. Moreover, the Sustainable Development Goals (SDGs) Agenda 2030 also serve as a strategy and plan to accomplish improved and building a more resilient and lasting future for all stakeholders. On that note, the purpose of this empirical study is to investigate the interactive influence of financial development along with natural resource rents on carbon emissions in the new BRICS economies for the period 1990–2019. The paper employed the panel data generalised least squares (GLS) technique. The panel-corrected standard error (PCSE) techniques were deployed for robustness purposes. To establish causality, this research utilised the Dumitrescu and Hurlin causality approach.

The GLS findings illustrate that there is a U-shaped association involving economic development and environmental quality. Most models also depict that both financial development of financial institutions and financial development of financial markets connections with emissions is significantly positive. As well, the paper noted no evidence of the financial environmental Kuznets curve (FEKC) hypothesis but a monotonic heightening relationship between financial development of financial markets and emissions. The study also spotlights that natural resource rents, energy consumption, and human capital create a positive and significant link with environmental degradation (dominantly just positive for technological innovation). On the other hand, this research outlines that the association between trade and carbon emissions is significantly negative (although just negative for FDI).

The interactions integrated in the regressions also generated informative outcomes. Initially, the interaction(s) involving financial development of financial institutions and financial development of financial markets with natural resource rent significantly mitigates emissions. Secondly, the interaction term (financial development of financial institutions, natural resource rent and financial development of financial markets) mix with trade significantly contributes to more carbon emissions (positively insignificant with energy use). Conversely, this parameter mix with human capital and technological innovation, respectively, is significantly negative (just negative for FDI).

The paper also utilised the Dumitrescu–Hurlin panel Granger causality to investigate direction of causality between and among the parameters. Therefore, the study shows that there is a unidirectional causality link from carbon emissions and/or economic growth and human capital. As well, the research ascertains a one-way causality from natural resource rents, energy consumption, and technological innovation to environmental degradation. The study also determined a bidirectional causality association between environmental quality and financial development of financial institutions, trade, and foreign direct investments. A unidirectional causality relationship runs from the interaction parameter (financial development of financial institutions and natural resource rents) to carbon emissions. In the same vein, a one-way causality link is produced from this interaction variable mix with either and/or FDI, trade, energy consumption, and technological innovation to environmental quality. Finally, a one-way causality connection running from environmental degradation to the interaction parameter (financial development of financial institutions, financial development of financial markets, natural resource rents and FDI) is produced.

This research has limitations that future research can be directed. Initially, the paper used total natural resource rents which are an aggregate measure. More studies could examine specific types of natural rents such as forest, mineral, oil, coal, and natural gas rents. Furthermore, this paper utilised carbon emissions as the measure of environmental quality. Future research could also examine other forms of environmental degradation such as ecological footprint and total greenhouse gas emissions and then compare findings. Finally, this paper only included the new BRICS countries. It is apparent that these economies have diverse green economy policies, emissions, financial systems, technological innovations, trade practices, natural resource rents, and also varied human capital, among others. On that note, country particular studies in the new BRICS are imperative to provide country particular recommendations along with corrective initiatives.

## Supplementary Information

Below is the link to the electronic supplementary material.

Supplementary file1 (XLSX 238 KB)

## Data Availability

The datasets used and/or analysed during the current study are available from the corresponding author on reasonable request.
